# Dominant-negative transforming growth factor-*β* receptor-armoured mesothelin-targeted chimeric antigen receptor T cells slow tumour growth in a mouse model of ovarian cancer

**DOI:** 10.1007/s00262-022-03290-6

**Published:** 2022-09-27

**Authors:** Ke Li, Jing Xu, Jing Wang, Chong Lu, Yilin Dai, Qing Dai, Wang Zhang, Congjian Xu, Shu Wu, Yu Kang

**Affiliations:** 1 Department of Obstetrics and Gynaecology, Shanghai Medical School, Fudan University, No. 419 Fangxie Road, Shanghai, 200011 China; 2 Nanjing Legend Biotechnology Co.,Ltd., 568 Longmian Avenue, Ltd. Life Science TechTown, Jiangning, Nanjing, 211100 China

**Keywords:** Ovarian cancer, Chimeric antigen receptor (CAR) T-cell therapy, Mesothelin (MSLN), Immunotherapy

## Abstract

Ovarian cancer is a major cause of death among all gynaecological cancers. Although surgery, chemotherapy and targeted therapy have yielded successful outcomes, the 5-year survival rate remains < 30%. Adoptive immunotherapy, particularly chimeric antigen receptor (CAR) T-cell therapy, has demonstrated improved survival in acute lymphoblastic leukaemia with manageable toxicity. We explored CAR T-cell therapy in a preclinical mouse model of ovarian cancer. Second-generation CAR T cells were developed targeting mesothelin (MSLN), which is abundantly expressed in ovarian cancer. Cytotoxicity experiments were performed to verify the lethality of CAR T cells on target cells *via* flow cytometry. The in vivo antitumour activity of MSLN CAR T cells was also verified using a patient-derived xenograft (PDX) mouse model with human tumour-derived cells. We also evaluated the potency of CAR T cells directed to MSLN following co-expression of a dominant-negative transforming growth factor-*β* receptor type II (dnTGF*β*RII). Our data demonstrate that anti-MSLN CAR T cells specifically eliminate MSLN-expressing target cells in an MSLN density-dependent manner. This preclinical research promises an effective treatment strategy to improve outcomes for ovarian cancer, with the potential for prolonging survival while minimizing risk of on-target off-tumour toxicity.

**Supplementary Information:**

The online version contains supplementary material available at 10.1007/s00262-022-03290-6.

## Introduction

Ovarian cancer is the second most common cause of gynaecologic cancer-associated death in women globally[1]. Despite the development of adjuvant therapies such as chemotherapy, targeted therapy, and immunotherapy, the 5-year survival for ovarian cancer patients has not markedly changed in most cases [2–4]. Among these therapies, immune checkpoint inhibitors including cytotoxic T-lymphocyte-associated protein 4, programmed cell death protein 1 (PD-1), and programmed death ligand 1 (PD-L1) have been approved for the treatment of a variety of tumours [5]. Ovarian cancer is an immunogenic tumour, and immunotherapy is a potential treatment for ovarian cancer [6], where the overall objective remission rate of anti-PD-1 antibody ranges from 10 to 15%, lower than that observed in other cancer types [7, 8]. This lower rate may be attributed to the low overall tumour mutational burden among the subtypes of ovarian cancer [9]. Therefore, development of new and effective treatments for ovarian cancer is necessary.

Owing to the key role of T cells in the immunosurveillance of ovarian cancer, adoptive T-cell therapy has received increasing attention as an immunotherapy strategy for ovarian cancer [10]. Patients treated with infiltrating T lymphocytes (tumour-infiltrating T lymphocytes [TILs]) exhibited a 5-year survival rate of 38.0%, which was significantly higher than that for patients without TILs (4.5%) [11] (*P* < 0.05). Additionally, one study showed that ovarian cancer patients that were administered with autologous TILs for treatment after surgery and chemotherapy exhibited a 3-year disease-free survival rate of 82.1%, significantly higher than that of the control group (54.5%) [12]. In another clinical study, seven patients with recurrent ovarian cancer were treated with autologous TILs; one patient exhibited complete tumour regression, while four showed more than 50% regression [13]. These results suggest that T-cell therapy can effectively inhibit the growth of ovarian cancer cells.

CAR T-cell therapy, one of the prominent immunotherapies, has shown impressive curative effect in malignant haematologic tumours [14, 15]. A series of CAR T-cell therapies are under preclinical and clinical evaluation and validation. Researchers have demonstrated robust clinical efficacy and tolerability in the treatment of leukaemia, lymphoma, and multiple myeloma using CAR T-cell therapy [16–19]. Although CAR T-cell therapy has shown promising results in haematologic tumours, its effectiveness in solid tumours is far from satisfactory. CAR T-cell therapy for solid tumours presents several obstacles and difficulties; it is more difficult for CAR T cells to migrate and infiltrate into solid tumours compared with haematologic tumours [20, 21]. The existence of an immunosuppressed tumour microenvironment (TME) in solid tumours is not conducive to the efficacy of CAR T cells. Particularly, the heterogeneity and antigen loss of solid tumours are often attributable for refractory solid tumour recurrence [22].

MSLN was originally identified by Pastan and colleagues as a tumour-associated antigen due to its limited expression by normal tissues and overexpression on tumours [23, 24]. Mature MSLN is a 40-kDa glycoprotein anchored at the cell membrane by a glycosylphosphatidylinositol linkage. MSLN is highly expressed on a variety of tumours, including ovarian cancer.

In our study, a humanized anti-MSLN single-domain antibody (clone ID is F3M) was fused to a 4-1BB co-stimulatory signalling domain and CD3 signalling domain to generate a second-generation CAR construct (designated as F3M CAR) targeting MSLN. We demonstrated the efficacy of F3M CAR T cells via both in vitro cytotoxicity assay and in vivo studies in mouse models. In order to resist the immunosuppressive effect of TGF-*β* in the solid tumour microenvironment, we generated LCAR-M23 by co-expression of a dominant-negative transforming growth factor-*β* receptor type II (dnTGF*β*RII) and F3M CAR. It was also demonstrated that dnTGF*β*RII conferred resistance to immunosuppressive effect of TGF-*β* on LCAR-M23 T cells, improved the functionality of CAR-T cells in the presence of TGF-*β*, and enhanced the efficacy of CAR-T cells in a mouse model as well. We've registered LCAR-M23 for clinical trials, and we are currently recruiting epithelial ovarian cancer patients (ClinicalTrials.gov: NCT04562298). Our encouraging preliminary results may help guide development of future clinical studies regarding CAR T-cell therapy.

## Materials and methods

### Patient samples

Tumour and blood samples were collected from patients at the Obstetrics and Gynaecology Hospital of Fudan University, Shanghai, China. The study was approved by the Institutional Research Ethics Committee of Obstetrics and Gynaecology Hospital of Fudan University, and written informed consent was obtained from each patient. The patients’ clinical and pathological characteristics are given in Table supplementary 1.

### Cell lines

Human embryonic kidney (HEK) 293 cells and CaOV-3 cells were maintained in DMEM (Sigma-Aldrich, USA) containing 10% foetal bovine serum (FBS) (Sigma-Aldrich, USA). OVCAR-3 cells, A-431 cells, K-563 cells, SKOV-3 cells, and OVCAR-8 cells were maintained in RPMI-1640 medium (Sigma-Aldrich, USA) containing 10% FBS. Different kinds of cells were obtained from the Zhang Zhigang lab and authenticated by STR (Short Tandem Repeat). All cells were periodically tested for mycoplasma. All kinds of cells were cultured in a cell incubator at 37 °C and 5% CO2.

### Preparation of lentivirus plasmids

The lentivirus packaging plasmid mixture was premixed with the transfer plasmid at a pre-optimized ratio with polyethylenimine, followed by transfection to HEK-293cells and overnight incubation. The virus-containing supernatants were collected and filtered through a 0.45 µm polyethersulphone filter, followed by ultra-centrifugation for lentivirus concentration. The virus pellets were rinsed with pre-chilled Dulbecco’s phosphate-buffered saline, after which the virus pellets were appropriately aliquoted and stored at − 80°C immediately.

### Collection and transduction of T lymphocytes

Leukocytes were collected from healthy donors by apheresis. Peripheral blood mononuclear cells (PBMCs) were isolated using Ficoll-Paque™ PLUS media (Global Life Sciences Solutions USA LLC) according to the manufacturer’s instructions. Human T cells were purified from PBMCs using the Pan T cell isolation kit (Miltenyi, Cat#130–096-535), following the manufacturer’s instructions. The purified T cells were subsequently pre-activated for 48 h using a human T cell activation/expansion kit (Miltenyi, Cat#130–091-441) according to the manufacturer’s instructions, and anti-CD3/CD28MACSiBead particles were added at a bead-to-cell ratio of 1:2. The pre-activated T cells were transduced using lentivirus stock in the presence of 7 µg/mL polybrene. The transduced cells were then transferred to a cell culture incubator for expression of the transgene under suitable conditions.

### Detection of MSLN expression in target cells

Target cells were incubated with rabbit anti-human MSLN antibody (Abcam, UK) or rabbit IgG as an isotype control (Abcam, UK). Alexa Fluor 647 AffiniPure goat anti-rabbit IgG (H + L) was used as the secondary antibody (Jackson ImmunoResearch Laboratories, USA). MSLN expression in target cells was analysed using a flow cytometer. Cells that were not stained with any antibody were labelled as PBS control; cells that were not stained with the first antibody, but were stained with the secondary antibody, were labelled as 2nd Ab control.

### Evaluation of in vitro activities of CAR T cells

#### In vitro cytotoxicity assay

Target cells were pre-stained using CellTrace Violet before co-culture with CAR T cells or untransduced T (UnT) cells. After incubating in a CO_2_ incubator for 20–24 h, the cells were collected and stained using 7-AAD. 7-AAD-positive cells from CellTrace Violet-positive cell population were gated using the FACS Celesta flow cytometer (BD Biosciences, USA). Cytotoxicity of CAR T cells was calculated as per the following formula: % Cytotoxicity = 100 × (% 7-AAD^+^ cells from co-cultures—% 7-AAD^+^ target cells only) / (100—% 7-AAD^+^ target cells only).

#### IFNγ and TNFα release assay

Additionally, the co-culture medium of effecter cells and target cells was collected to determine secretions of the cytokines IFN*γ* and TNF*α* using cytokine kits (Cisbio).

#### Long-term co-culture assay by repetitive stimulation

On day 0 (5 days after transduction), 2 × 10^5^ OVCAR-8 cells were plated in a 24-well plate to establish a monolayer. T cells were counted, and 0.4 × 10^4^ viable CAR T cells were plated on top of the OVCAR-8 cells in fresh media with or without 5 ng/mL of human transforming growth factor beta 1 (TGF-*β*1). On day 3, the percentage of CD3^+^ T cells and that of CAR T cells in total live cells were determined via flow cytometry analysis. New 2 × 10^5^ OVCAR-8 cell monolayers were plated on day 0, and 0.4 × 10^4^ CAR T cells which expanded in wells (i.e. the cell density indicated the minimum number of cells in the wells required for further studies) were replated on the new monolayer as on day 0. The process was repeated for 3 rounds of stimulations. Fold expansion after each stimulation was calculated as [viable CAR T cells after co-culture / viable CAR T cells that were seeded into the co-culture medium]. Cumulative fold expansion was determined using the formula [(fold expansion in 1st round) × (fold expansion in 2nd round) × (fold expansion in 3rd round)].

### Design of the CAR construct

F3M CAR consisted of a humanized anti-MSLN single domain antibody (Clone F3M) with a CD8*α* hinge, and intracellular T cell signalling (CD3 zeta) and a co-stimulatory (4-1BB) domain. dnTGF*β*RII was a truncated TGF-*β* receptor II that lacked the intracellular domain necessary for downstream signalling. LCAR-M23 cells were designed to target and eliminate malignant tumour cells expressing MSLN and to simultaneously express dnTGF*β*RII to reduce immunosuppression of T cells by TGF-*β* in tumours. The expression of F3M CAR and dnTGF*β*RII components was driven by a single human elongation factor 1 alpha (hEF1*α*) promoter and separated by two P2A (porcine teschovirus-1 2A) peptide-encoding sequences (Fig. 2).

### Western blotting for SMAD2 and pSMAD2

LCAR-M23 CAR T cells and F3M CAR T cells were treated with or without 5 ng/mL of human TGF-*β*1 for 45 min at 37 °C after being cultured overnight in a CO_2_ incubator. 5 × 10^6^ CAR T cells were centrifuged at 2500 × *g* for 5 min, subjected to washing steps twice in cold PBS, and resuspended in 0.2 mL of radio immunoprecipitation assay buffer with 10 μL/mL of Halt Protease and Phosphatase Inhibitor Cocktail (Abcam, UK). The mixture was incubated on ice for 15 min and centrifuged at ~ 14,000 × *g* for 10 min. Supernatants were harvested for conducting western blotting experiments. Protein sample (cell lysate) was mixed with 5 × non-reducing (NR) or 5 × reducing (R) loading buffer at a 4:1 ratio (V/V). The samples were heated for 5 min. Thirty µg of proteins were loaded on a 4%–20% gradient ExpressPlus™ PAGE Gel (GenScript, USA). After transfer of the protein bands onto nitrocellulose membranes, proteins were probed with a rabbit antibody against SMAD2 (86F7) (60 kDa) and a rabbit antibody against phospho-SMAD2 (Ser465/467) (60 kDa) at concentrations suggested by the manufacturer (Cell Signalling Technology, USA). Membranes were then exposed to peroxidase-conjugated secondary antibodies and protein expression was quantitated via chemiluminescence detection (LumiGLO, Cell Signalling Technology). Blots were stripped and re-probed with rabbit anti-*β* Actin (45 kDa; (Cell Signalling Technology, USA) to confirm equal protein loading.

### Immunohistochemistry

Paraffin-embedded tissue samples were sliced into 4 mm thickness. Antigen retrieval was performed by a pressure cooker for 15 min in EDTA Antigen Retrieval Solution (Solarbio, Beijing, China) according to the instructions. Specimens were incubated with antibodies specific for CD45 (1:300, Cell Signalling Technology, USA), CD4(1:300, Abcam, UK), CD8*α* (1:300, Cell Signalling Technology, USA), Foxp 3(1:250, Abcam, UK) overnight at 4 ℃. Following incubation with the secondary antibody at room temperature for 30 min, the immunodetection was performed by using the DAB Horseradish Peroxidase Colour Development Kit (Beyotime Biotechnology, Shanghai, China) according to the instructions.

At least three fields were selected randomly for quantitative analysis. The following immunohistochemistry score (range 0–8) assigned to the sections: a. the percentage of positive cells in the tissue:0 = no staining, 1 = 1–10%, 2 = 11–25%, 3 = 26–50%, 4 = 51–75%, 5 = 76–100%; b. the staining intensity was scored on a scale of 0–3: 0, negative; 1, weak; 2, moderate; and 3, strong. The immunohistochemistry score = a + b.

### Evaluation of CAR T cells in xenograft mouse models

Antitumour activity of CAR T cells was assessed in vivo in an OVCAR-3 xenograft mouse model, and PDX models. 10 × 10^6^ OVCAR-3 cells, 10 × 10^6^ OVCAR-8 cells, or patient-derived ovarian tumours were implanted subcutaneously on day 0 in immunocompromised NOD-Prkdcem26Il2rgem26Nju (NCG) mice. Once the tumours attained a volume of 120–150 mm^3^, the mice were randomized into treatment groups. CAR T cells were administered intravenously. Body weight of mice and tumour volume were monitored twice a week. Peripheral blood samples were collected from mice once a week to determine the proliferation of CAR T cells in vivo via flow cytometry analysis.

### Statistical analysis

Statistical analyses were carried out using GraphPad Prism 9(GraphPad Software, Inc., USA). The information about statistical details is indicated in the figure legends or text. All the data were expressed as the mean ± standard error of the mean (SEM) or standard deviation (SD) and were analysed using two-tailed unpaired Student’s t test or two-way analysis of variance test.

## Results

### Expression of MSLN in ovarian carcinoma

**Fig. 1:**
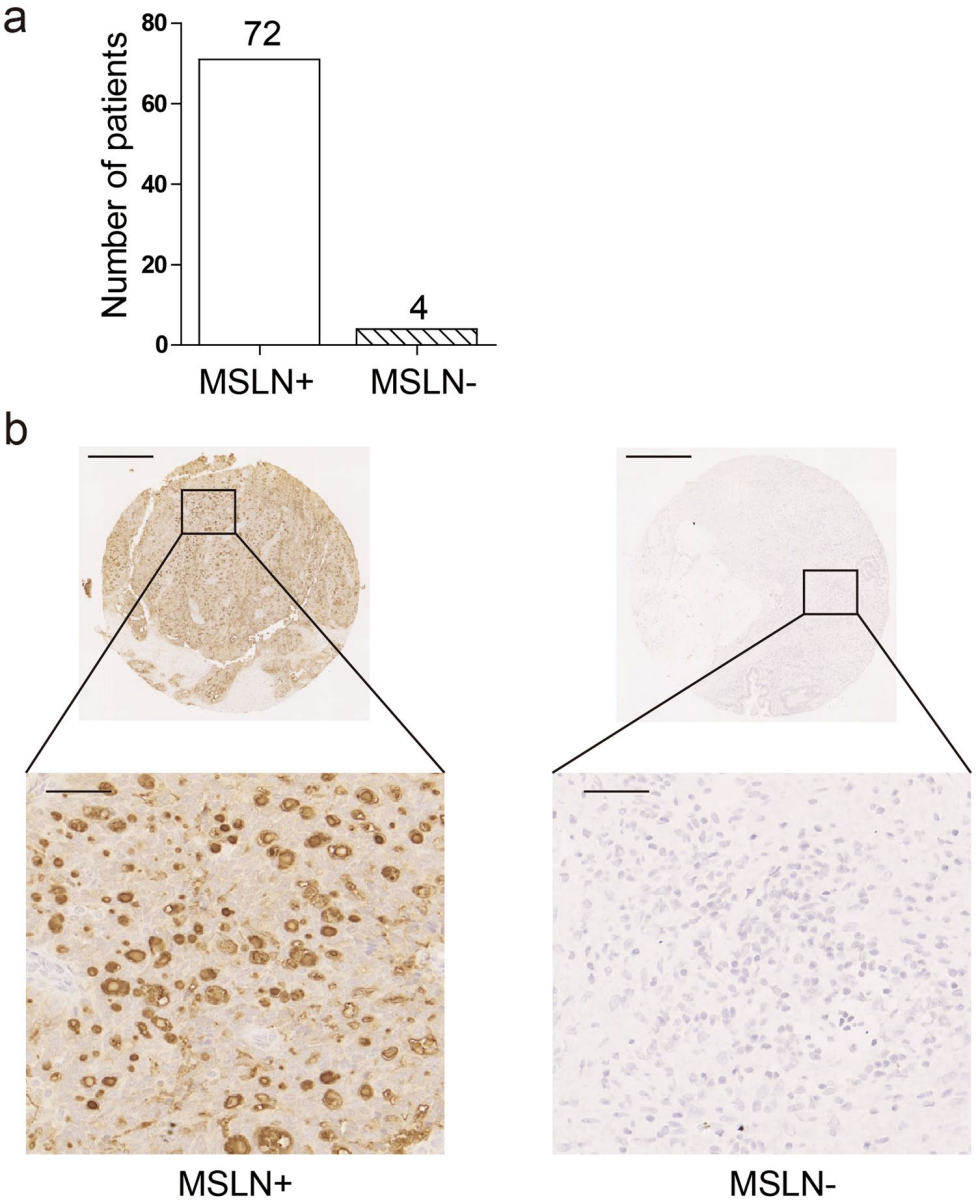
Expression of MSLN in ovarian carcinoma(*n* = 76). **a** Measurement of expression of MSLN in tissue microarray of ovarian cancer determined by immunohistochemistry. **b** Representative images (50 × and 400 ×) of MSLN expression. Scale bar represents 500 µm

MSLN has been shown to be overexpressed in mesotheliomas, pancreatic cancers, ovarian cancers, and certain lung cancers and is not expressed in normal human tissues except mesothelial cells at relatively low levels [25]. A tissue microarray containing 76 human ovarian cancer samples was stained using immunohistochemistry to analyse the expression of MSLN in the samples (Fig. 1a and b). Among the 76 samples analysed, 72 (94.7%) were MSLN-positive, confirming that MSLN was widely expressed in human ovarian cancer samples.

### In vitro cytotoxicity of LCAR-M23 CAR T cells

**Fig. 2:**
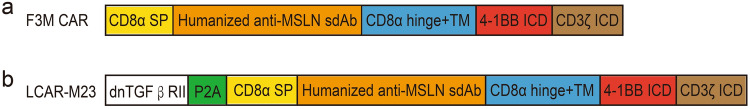
Diagrams of F3M CAR and LCAR-M23. **a** Diagram of F3M CAR is shown. F3M CAR consists of a human CD8*α* SP, humanized anti-MSLNsdAb (clone F3M), a human CD8*α* hinge + TM domain, a human 4-1BB cytoplasmic domain, and a human CD3ζ cytoplasmic domain. **b** Diagram of LCAR-M23 is shown. LCAR-M23 consists of dnTGF*β*RII and F3M CAR CD3*ζ*, cluster of differentiation 3 zeta; CD8*α* hinge + TM, cluster of differentiation 8 alpha hinge and transmembrane; CD8*α* SP, cluster of differentiation 8 alpha signal peptide; dnTGF*β*RII, dominant-negative transforming growth factor-*β* receptor type II; ICD, intracellular domain; MSLN, mesothelin; sdAb, single-domain antibody

**Fig. 3:**
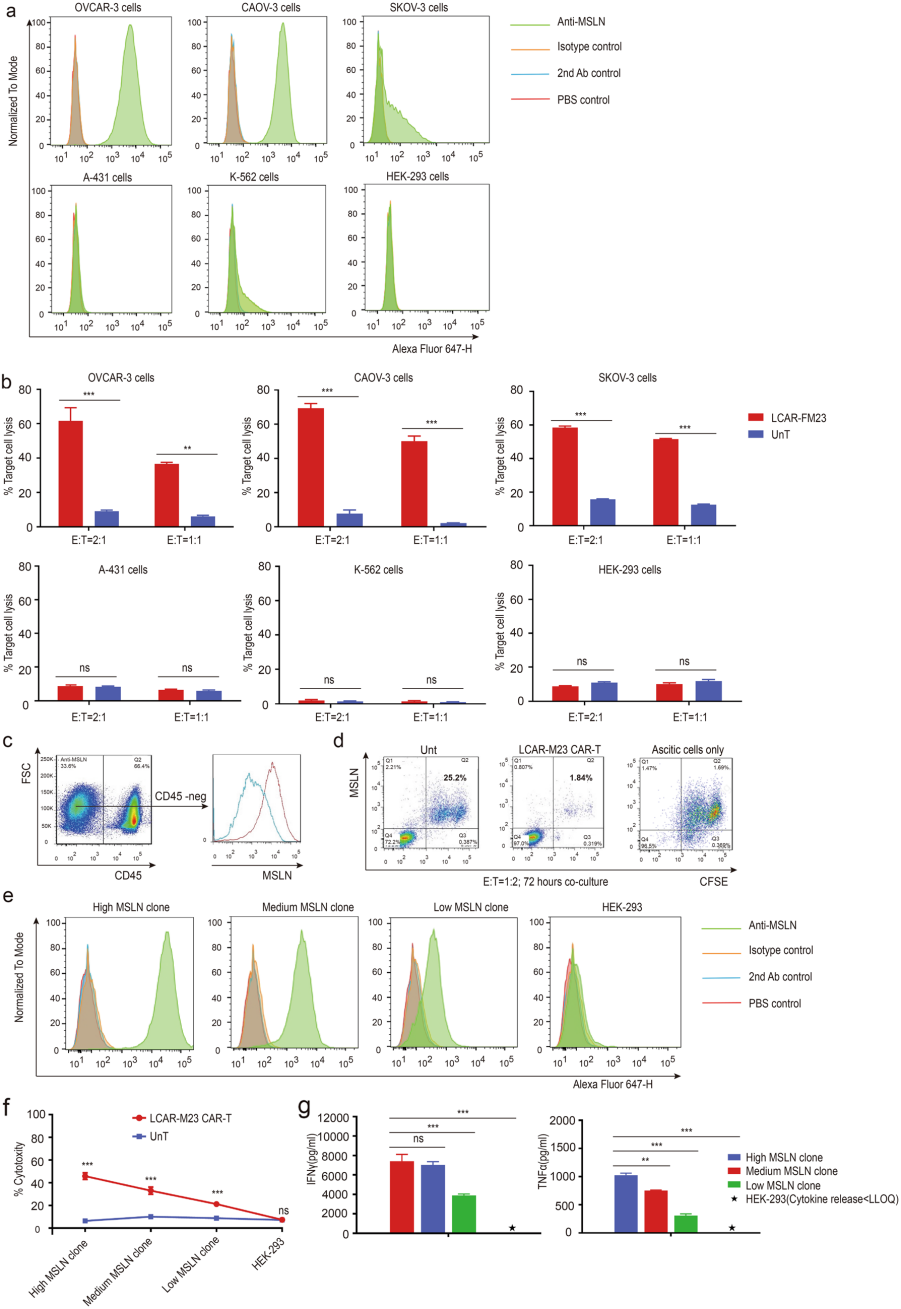
In vitro cytotoxicity of LCAR-M23 CAR T cells. **a** MSLN expression in six kind of cell lines was determined by flow cytometry. MSLN is expressed in OVCAR-3 cells, Caov-3 cells, and SK-OV-3 cells; MSLN is not expressed in A-431 cells, K-562 cells, and HEK-293 cells. **b** In vitro cytotoxicity of LCAR-M23 CAR T cells againstOVCAR-3 cells, Caov-3 cells, SK-OV-3 cells, A-431 cells, K-562 cells and HEK-293 cells. **c** Flow cytometry analysis of MSLN expression in ascitic cells of ovarian cancer patients. **d** In vitro cytotoxicity of LCAR-M23 CAR T cells against autologous ascitic tumour cells was determined by flow cytometry. **e** MSLN expression in engineered HEK-293 cell lines was determined by flow cytometry. **f** Cytotoxicity of LCAR-M23 CAR T cells against HEK-293 cells expressing gradient levels of MSLN. **g** IFN*γ* and TNF*α* release of LCAR-M23 CAR T cells in co-culture with HEK-293 cells expressing gradient levels of MSLN by using cytokine kits. The level of IFN *γ* and TNF *α* release of high MSLN clone LCAR-FM23 was significantly increased compared with only HEK-293 group, medium MSLN clone group and low MSLN clone group. Data shown in **a**–**g** are at least 3 experiments (mean ± SEM). *p < 0.05 ** *p* < 0.01, *** *p* < 0.001

F3M CAR and LCAR-M23 T cells were designed for the following in vivo and in vitro antitumour experiment (Fig. 2). We determined MSLN expression in different cell lines. As shown in Fig. 3a, MSLN expression was detected in ovarian cancer cell lines, OVCAR-3, CAOV-3, and SK-OV-3, whereas no MSLN expression was found in A-431, K-562, and HEK-293 cell lines (Fig. 3a). To assess the specific cytotoxicity of CAR T cells, LCAR-M23 CAR T cells were co-cultured with the above-mentioned 6 cell lines for a period of 20–24 h, and the cytotoxicity was determined via flow cytometry analysis. Results (Fig. 3b) showed that LCAR-M23 CAR T cells possessed specific killing property against MSLN-expressing ovarian cancer cell lines (OVCAR-3, CAOV-3, and SK-OV-3), and no cytotoxicity was observed against MSLN-negative cell lines (A-431, K-562, and HEK-293).

To further assess the functionality of LCAR-M23 cells in a close-to-clinical setting, the cytotoxicity of LCAR-M23 CAR T cells prepared from PBMCs of ovarian cancer patients against autologous ascitic cells was evaluated. Erythrocytes were removed from ascites and MSLN expression was measured in CD45-negtive cell population from the ascitic cells. As shown in Fig. 3c, MSLN was expressed in CD45-negative ascitic cells. Ascitic cells were pre-stained with carboxyfluoresceinsuccinimidyl ester (CFSE) before co-culture with LCAR-M23 CAR T cells at an effecter to target (E:T) ratio of 1:2 for 3 days. Patient T lymphocytes (UnT) were co-cultured with CFSE-stained ascitic cells under the same conditions as the negative control. The cytotoxicity was analysed via flow cytometry analysis. As shown in Fig. 3d, LCAR-M23 CAR T cells displayed remarkable lethality such that the percentage of MSLN-positive ascitic cells decreased to 1.8% compared to 25.2% in the UnT group.

To investigate the correlation of cytotoxicity of LCAR-M23 with MSLN density, HEK-293 cell lines were stably transfected with human MSLN to establish a panel of cell lines expressing gradient levels of MSLN. As shown in Fig. 3e, three engineered HEK-293 cell lines with gradient MSLN expression levels were obtained, namely low-MSLN clone, medium-MSLN clone, and high-MSLN clone. LCAR-M23 CAR T cells were co-cultured with these 3 cell lines and HEK-293 cells. The cytotoxicity of CAR T cells was determined via flow cytometry analysis, whereas release of IFN*γ* and TNF*α* by CAR T cells was detected using homogeneous time-resolved fluoroimmunoassay. As shown in Fig. 3f and g, cytotoxicity, IFN*γ* release, and TNF*α* release of LCAR-M23 CAR T cells were dependent on MSLN density.

### Evaluation of in vivo efficacy of LCAR-M23 CAR T cells

**Fig. 4:**
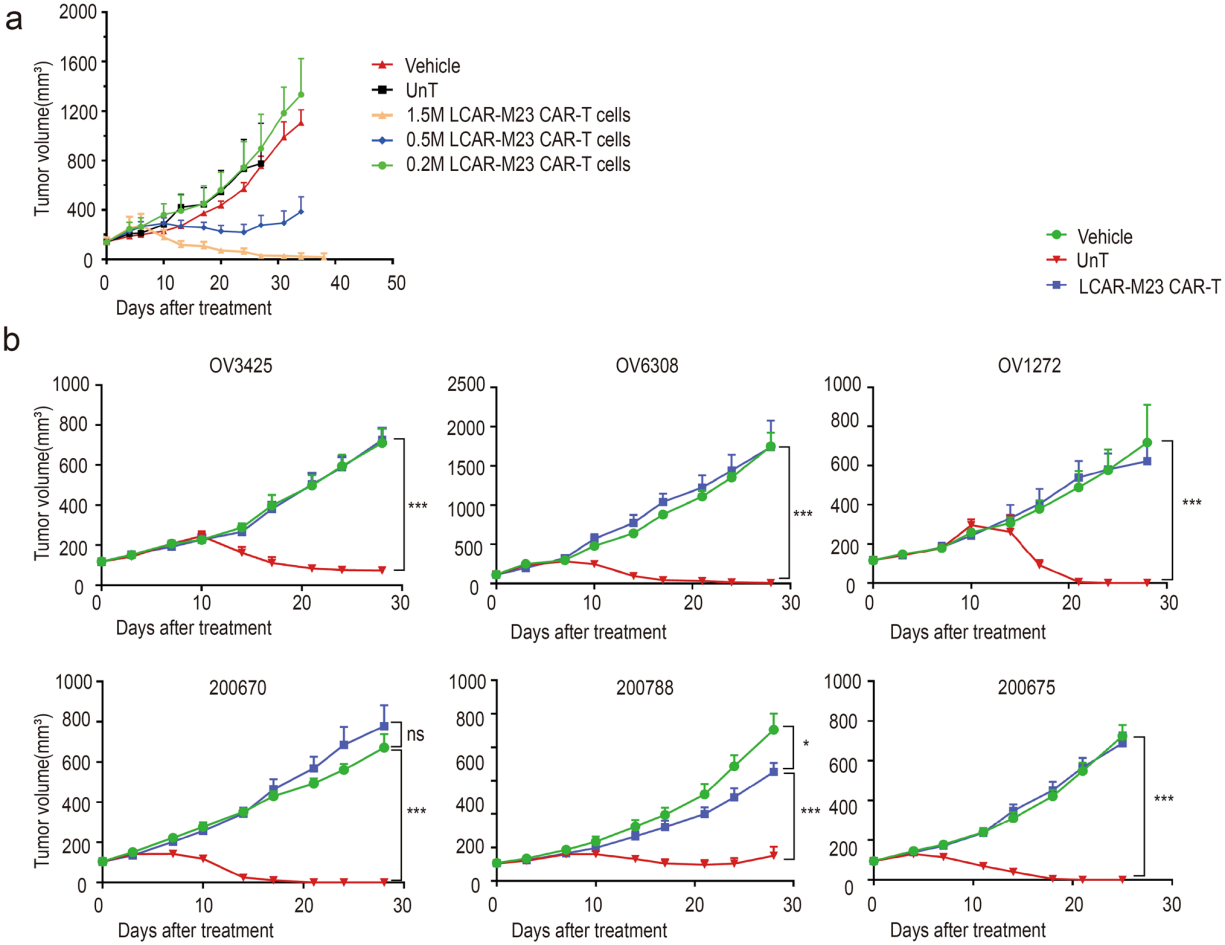
Evaluation of in vivo efficacy of LCAR-M23 CAR T cells. **a** The tumour-bearing mice were randomly assigned to five groups. Tumour growth from dose escalation of LCAR-M23 CAR T cells in an OVCAR-3 xenograft mouse model. **b** Tumour growth from vector control, UnT control and LCAR-M23 CAR T treatment in the PDX models of ovarian carcinoma. LCAR-M23 CAR T cells significantly reduced ovarian cancer burden in the PDX models. Data shown in **a**–**b** are at least 3 mice in each group (mean ± SEM). *p < 0.05 ** *p* < 0.01, *** *p* < 0.001

Next, we evaluated the in vivo efficacy and antitumour activity of LCAR-M23 CAR T cells in an OVCAR-3 xenograft mouse model. 10 × 10^6^ OVCAR-3 cells were implanted subcutaneously on day 0 in immunocompromised NCG mice. Once tumours reached a volume of approximately 150 mm^3^, the mice were randomized into three treatment groups, wherein 1.5 × 10^6^, 0.5 × 10^6^, and 0.2 × 10^6^ LCAR-M23 CAR T cells were administered intravenously, respectively. UnT cells and vehicle were injected as negative controls for LCAR-M23 CAR T cells. Tumour volume was determined twice a week. As shown in Fig. 4a, a high dose of LCAR-M23 CAR T cells efficiently eradicated tumours in mice, rendering them tumour-free; a medium dose of LCAR-M23 CAR T cells inhibited tumour growth, whereas a low dose of LCAR-M23 CAR T cells did not result in tumour inhibition activity compared to that observed in UnT and vehicle groups. Thus, the antitumour efficacy of LCAR-M23 CAR T cells in OVCAR-3 xenograft mouse model was found to be dose-dependent.

The antitumour efficacy of LCAR-M23 CAR T cells was further evaluated in a panel of PDX models. Immunocompromised NCG mice were subcutaneously inoculated with ovarian cancer patient-derived tumours. When tumour volume reached a volume of approximately 120 mm^3^, the tumour-bearing mice were treated with LCAR-M23 CAR T cells. A group of mice each was treated with Hank’s balanced salt solution (HBSS) and UnT cells as negative controls for LCAR-M23 CAR T cells. Tumour volume was monitored twice a week. CAR T cells failed to inhibit tumour growth in the OV0276 model, which was an MSLN-negative PDX model (Supplementary Fig. S1a and S1b). Notably, LCAR-M23 CAR T cells efficiently eradicated tumours or inhibited tumour growth in all 6 MSLN-positive PDX models (Fig. 4b).

### dnTGF*β*RII confers resistance to TGF-*β* in LCAR-M23 CAR T cells

**Fig. 5:**
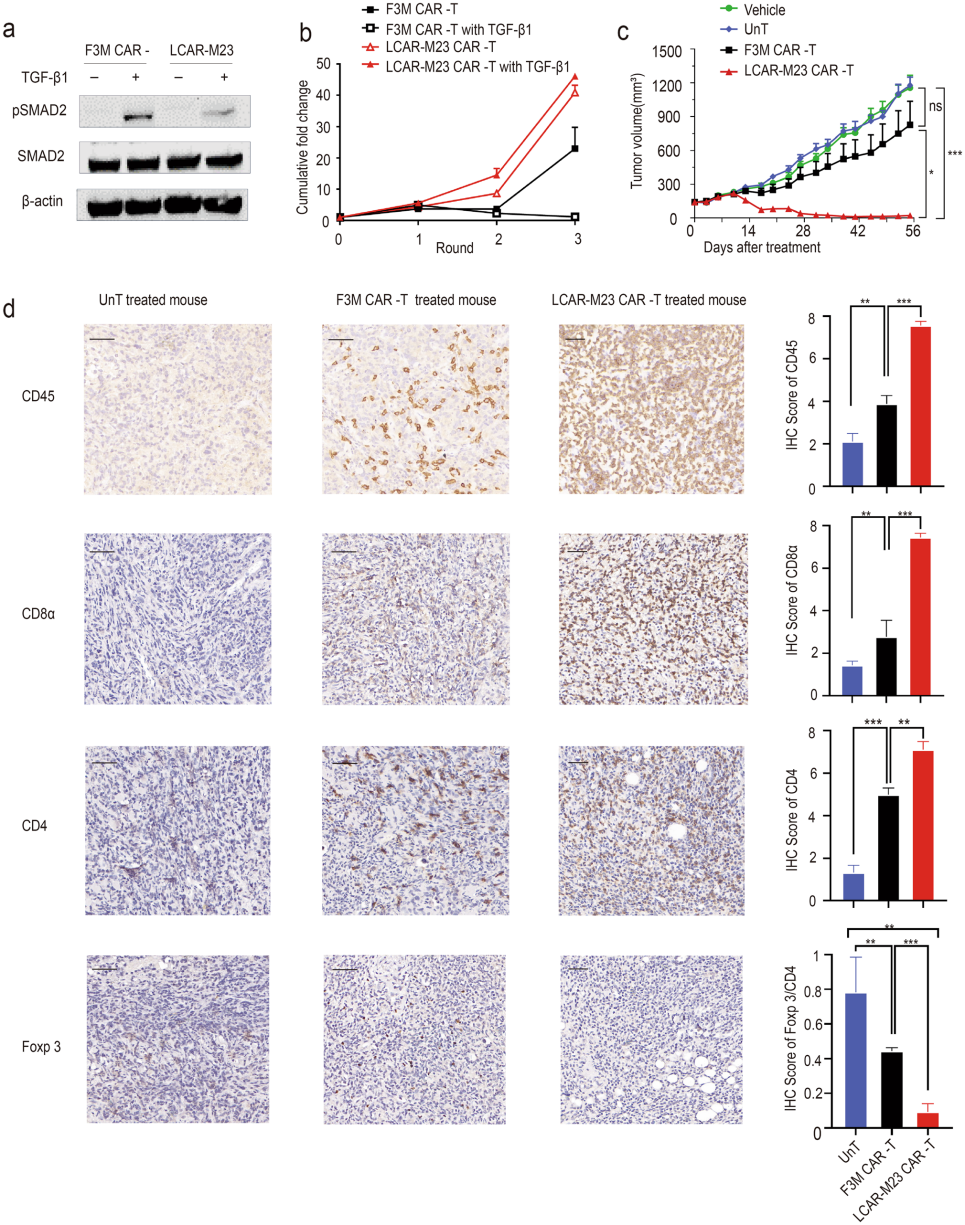
dnTGF*β*RII confers resistance against TGF-*β* in LCAR-M23 CAR T cells. **a** Western blotting of SMAD2 and pSMAD2 expression in LCAR-M23 CAR T cells and F3M CAR T cells. Results showed that dnTGF*β*RII inhibited phosphorylation of SMAD2. **b** Proliferation of LCAR-M23 CAR T cells and F3M CAR T cells in a long-term co-culture assay. **c** Tumour growth curve based on data obtained after analysis using the OVCAR-8 xenograft mouse model. LCAR-M23 CAR T cells exhibited more potent antitumour efficacy than F3M CAR at a suboptimal dose. **d** Distribution of T cells in the tumour of 3 groups determined by IHC staining with anti-human CD45 antibody, anti-human CD4 antibody, anti-human CD8*α* antibody, anti-human Foxp 3 antibody CAR, chimeric antigen receptor; dnTGF*β*RII, dominant-negative transforming growth factor-*β* receptor type II; MSLN, mesothelin; SMAD2, mothers against decapentaplegic homolog 2; pSMAD2, phosphorylated SMAD2, TGF-*β*1, transforming growth factor beta 1; UnT, untransduced T cells; IHC, immunohistochemistry. *n* = 3 each group, × 400 magnification, scale bar represents 50 μm. Quantification of CD45, CD8, CD4 and Foxp 3/CD4 score of all groups by the criterion has been described in the methods. Error bars represent mean ± SEM. *p < 0.05 ** *p* < 0.01, *** *p* < 0.001

Studies have shown that increased serum TGF-*β* levels are associated with ovarian cancer metastasis and drug resistance, and that dnTGF*β*RII helps CAR T cells resist the immunosuppressive effect of TGF-*β* [26, 27]. Cell lysate of LCAR-M23 CAR T cells and F3M CAR T cells pretreated with or without TGF-*β*1 were subjected to western blotting assay to probe mothers against decapentaplegic homolog 2 (SMAD2) and phosphorylated SMAD2 (pSMAD2). Expression of the housekeeping gene *β*-actin was used as internal reference. The results demonstrated that dnTGF*β*RII conferred resistance to LCAR-M23 CAR T cells against TGF-*β* signalling (Fig. 5a).

LCAR-M23 cells were targeted and eliminated malignant tumour cells expressing MSLN and simultaneously expressed dnTGF*β*RII to reduce immunosuppression of T cells by TGF-*β* in tumours. To assess the biological function of dnTGF*β*RII, we compared the expansion potential of LCAR-M23 CAR T cells and F3M CAR T cells in a long-term co-culture assay through repetitive challenge by OVCAR-8 cells in the presence or absence of human TGF-*β*1. As shown in Fig. 5b, after 3 rounds of co-culture, LCAR-M23 CAR T cells exhibited excellent proliferation irrespective of the presence of TGF-*β*. In contrast, TGF-*β* significantly inhibited the proliferation of F3M CAR-T cells. These results demonstrated that dnTGF*β*RII transgene conferred resistance to TGF-*β* signalling in LCAR-M23 CAR T cells and enhanced the activities of CAR T cells in the presence of TGF-*β*.

The antitumour activity of F3M CAR T cells and LCAR-M23 CAR T cells was further evaluated in the OVCAR-8 xenograft mouse model. 1 × 10^7^ OVCAR-8 cells were implanted subcutaneously in NCG mice. Once tumours reached a volume of approximately 120 to 150 mm^3^, the mice were randomized into treatment groups (n = 5 in each group). 0.1 × 10^6^ CAR-positive T cells (a suboptimal dose) in a 200 µL dose were administered intravenously (day 0). HBSS medium containing no T cells and UnT cells were used as negative controls. Tumour volume and body weight were measured twice a week. Percentages of CD3^+^ T cells and MSLN CAR T cells in live peripheral blood cells were determined via flow cytometry analysis once a week. As shown in Fig. 5c, LCAR-M23 CAR T cells demonstrated better antitumour efficacy than F3M CAR T cells in vivo. Compared to the vehicle control group and UnT group, F3M CAR T cells demonstrated no better antitumour efficacy. No tumour-inhibitory effect was observed by infusion of Vehicle or UnT. Proliferation of CD3^+^ T cells and MSLN CAR T cells was greater in the cohort treated with LCAR-M23 CAR T cells than in the cohort treated with F3M CAR T cells. Populations of CD3^+^ T cells and MSLN CAR T cells declined 5 weeks after infusion of CAR T cells (Supplementary Fig. S2a and S2b). Moreover, LCAR-M23 CAR T cells reduced tumour burden in OVCAR-8 tumour-grafted NCG mice with no significant change in body weight compared with cohorts treated with vehicle or UnT (Supplementary Fig. S2c). The results demonstrated that dnTGF*β*RII transgene improved the in vivo efficacy of CAR T cells in mice bearing xenograft tumours. Furthermore, satellite groups were designed and mice were euthanized 14 days after T cell administration. Their tumours were resected, fixed with formalin, and embedded in paraffin. The tumour sections were stained with anti-human CD45 antibody, anti-human CD4 antibody, anti-human CD8*α* antibody, anti-human Foxp 3 antibody and the distribution of T cells in tumours was immunohistochemically investigated. As shown in Fig. 5d, this analysis revealed an increased infiltration of CD45^+^ T cells, CD4 + T cells and CD8 + T cells were observed in the tumours of mice treated with LCAR-M23 CAR T cells, compared to those of mice treated with F3M CAR T cells or UnT cells. And for the FOXP3 + regulatory T cells, it demonstrating a significantly decreased infiltrating diversity in the tumour of mice treated with LCAR-M23 CAR T cells. These results indicated that dnTGF*β*RII improved the tumour infiltration of CAR T cells.

## Discussion

Until recently, FDA has approved four CD19-targeting CAR T-cell therapy against haematologic malignancies, including acute lymphoblastic leukaemia in 2017, large B cell lymphoma in 2017, mantle cell lymphoma in 2020, and relapsed or refractory large B cell lymphoma in 2021. However, CAR-T cell therapy counteracting solid tumour is far from satisfactory, mainly attributable to the heterogeneity and uneven expression of antigens in solid tumours. As a result, there hasn’t been a particular antigen which characteristics is equivalent enough to be directed for solid tumour until now. Additionally, in vivo TME is usually an immunosuppressed milieu due to multiple local alterations contrasting haematologic malignancies, which impair the tumouricidal effect of infiltrated lymphocytes as well as CAR-T cells [28]. Therefore, the selection and optimization of effective and highly specific antigen target and the reversal of immunoinhibitory features in immune microenvironment to some extent may overcome the obstacle to serve a promising design of a successful CAR T-cell therapy against solid tumour.

Mesothelin, a tumour differentiation antigen, has been shown to be overexpressed in human ovarian cancer [29]. Serum mesothelin levels have been found to be elevated in a majority of ovarian cancer patients (67%) [24]. Notably, studies have shown that mesothelin may play a key role in the adhesion and implantation of ovarian cancer cells into the peritoneal cavity [24]. These data suggest that mesothelin may be on the top of CAR-T cell targets for in ovarian cancer immunotherapy. Clinically, few trials investigating CAR T-cell treatments for ovarian cancer are under way, with common targets including MSLN, FSHR, MUC16, and HER2. The results of one such clinical trial targeting MSLN in ovarian cancer have been published. In this phase 1 trial (NCT02159716), six patients with recurrent serious epithelial ovarian cancer were recruited and administered with autologous CAR T cells prepared via lentivirus transduction. This second-generation CAR construct consisted of murine anti-MSLNscFvSS1 and 4-1BB/CD3. Four patients received a dose of 3 × 10^7^cells/m^2^, and two received a dose of 3 × 10^8^ cells/m^2^. No acute AE or cytokine release syndrome was observed, and all six patients showed stable disease state. CAR T-MSLN cells were administered and expanded in the blood of all patients and migrated to the tumour sites, resulting in clearance of malignant cells in one patient [18].

In our study, we applied the LCAR-M23 technology to genetically modify autologous peripheral blood T cells to enable recognition and elimination of ovarian cancer cells with high expression of MSLN. MSLN target area was humanized sdAb F3M (cloning) for MSLN specificity recognition and CD137(4-1BB) & CD3ζ intracellular signal transduction area fused, for the initiation of downstream signalling of T cell activation. MSLN-targeted CAR T-cell therapy in PDX mouse models has been shown to be effective in mesothelioma, but efficacy has not been reported in ovarian cancer [30]. In vitro and in vivo studies have confirmed that LCAR-M23 has a targeted effect on MSLN-expressing human ovarian cancer cells. Moreover, the eradication of LCAR-M23 is MSLN density dependent, translating into strong cytotoxicity to tumour cells with high expression levels of MSLN and weak or no cytotoxicity to cells with low expression levels of MSLN. Our results showed that CD3^+^ T cells were not detected in the liver and lungs of mice treated with LCAR-M23 CAR T cells and F3M CAR T cells, indicating that CAR-T cells were not recruited to healthy organs like the liver and lungs, which indicates the safety and low side effect of this method.

In a complex TME, the survival, proliferation, and activation of CAR T cells are affected by a variety of immunosuppressive cells and factors [31], among which TGF-*β* is a key regulator in reshaping TME due to vast roles in tumour mediated immune suppression. First, elevated secretion of TGF-*β* is commonly observed in lots of malignancies, including both cancerous and stromal cells, such as carcinoma-associated fibroblasts (CAFs), vascular endothelial cells (VECs), and mesenchymal stem cells (MSCs) [31–33]. Second, TGF-*β* treatment attenuates anti-tumour response mediated by T cell, NK cell and macrophage[34]. Since several studies have shown that increased serum TGF-*β* levels are associated with metastasis and drug resistance in ovarian cancer, we assume that targeting TGF-*β* signalling by a dominant-negative TGF-*β* receptor on CAR-T cells as a decoy appears an attractive approach to block TGF-*β* intracellular signal transduction and therefore enhance anti-tumour efficacy of MSLN-directed CAR T cell. To be mentioned, there are also many trials which focus on the evaluation of TGF-*β* inhibitors for cancer therapy, including small molecular antagonist targeting intracellular kinase, neutralization antibody blocking endogenous TGF-*β* or TGF-*β* receptor, TGF-*β* or TGF-*β* receptor silence or knock-out, and unique disruptors for TGF-*β*-TGF-*β* receptor ligation [35–37]. However, given the multiple aspects of TGF-*β* in immune homeostasis, blockade of TGF-*β* systemically has a high chance leading to more side effects. Therefore, the cell-intrinsic and specific blockade of TGF-*β* just in CAR-T cells, like dnTGF*β*RII, is preferred. As we expected, we found that CAR-T cells with transduced dnTGF*β*RII showed insensitive to TGF-*β* due to less phosphorylated SMAD2 and therefore robust killing efficacy in vivo*.*

In summary, the efficacy of LCAR-M23 was evaluated in mouse models. LCAR-M23 T cells failed to inhibit tumour growth in the MSLN-negative PDXOV0276 model. Impressively, LCAR-M23 T cells efficiently eradicated tumours in all 6 MSLN-positive PDX models. Furthermore, it was also demonstrated that dnTGF*β*RII conferred resistance to the immunosuppressive effects of TGF-*β* on LCAR-M23 T cells, improved the functionality of CAR T cells in the presence of TGF-*β*, and enhanced the efficacy of CAR T cells in a xenograft model as well. These encouraging preliminary results can guide the development of improved clinical studies of safe, powerful, and feasible CAR T-cell therapy in the future.

### Supplementary Information

Below is the link to the electronic supplementary material.

Supplementary file1 (DOCX 13 KB)

Supplementary file2 (TIF 2494 KB)

Supplementary file3 (TIF 1244 KB)

## Abbreviations

AEs: Adverse events
CART: Chimeric antigen receptor T
CFSE: Carboxyfluoresceinsuccinimidyl ester
dnTGF*β*RII: Dominant-negative transforming growth factor-*β* receptor type II
HBSS: Hank’s balanced salt solution
IHC: Immunohistochemistry
MSLN: Mesothelin
PBMCs: Peripheral blood mononuclear cells
PD-1: Programmed cell death protein 1
PD-L1: Programmed death ligand 1
PDX: Patient-derived xenograft
TGF-*β*1: Transforming growth factor beta 1
TILs: Tumour-infiltrating T lymphocytes
TME: Tumour microenvironment
UnT: Untransduced T cells
