# Correction to: Dominant‑negative transforming growth factor‑*β* receptor‑armoured mesothelin‑targeted chimeric antigen receptor T cells slow tumour growth in a mouse model of ovarian cancer

**DOI:** 10.1007/s00262-024-03668-8

**Published:** 2024-04-18

**Authors:** Ke Li, Jing Xu, Jing Wang, Chong Lu, Yilin Dai, Qing Dai, Wang Zhang, Congjian Xu, Shu Wu, Yu Kang

**Affiliations:** 1 Department of Obstetrics and Gynaecology, Shanghai Medical School, Fudan University, No. 419 Fangxie Road, Shanghai, 200011 China; 2 Nanjing Legend Biotechnology Co., Ltd., 568 Longmian Avenue, Ltd. Life Science TechTown, Jiangning, Nanjing, 211100 China

**Correction to: Cancer Immunology, Immunotherapy (2023) 72:917–928** 10.1007/s00262-022-03290-6

In the original version of this article, Ke Li and Jing Xu should have been denoted as equally contributing authors.

