# Supplementary material for: Dominant-negative transforming growth factor-β receptor-armoured mesothelin-targeted chimeric antigen receptor T cells slow tumour growth in a mouse model of ovarian cancer
Source: Cancer Immunol Immunother. 2022 Sep 27;72(4):917–28. doi: 10.1007/s00262-022-03290-6 (PMC10025183; doi:10.1007/s00262-022-03290-6)
Supplement: Supplementary file 1 — Supplementary file1 (DOCX 13 KB) [file 262_2022_3290_MOESM1_ESM.docx]

Table S1: Clinical and pathological characteristics of the ovarian cancer patients（n=76）

| Characteristics | |
| --- | --- |
| Age(years) | 55 (32-81) |
| Stage(FIGO 2017) |  |
| I | 14 |
| II | 4 |
| III | 45 |
| IV | 13 |
| Histopathological subtype |  |
| High-grade adenocarcinoma | 70 |
| Low-grade adenocarcinoma | 1 |
| Endometrioid adenocarcinoma | 1 |
| Clear cell carcinoma | 2 |
| Mucinous epithelial ovarian carcinoma | 1 |
| Ovarian sarcoma | 1 |

Notes: a. Ages are presented as median(minimum-maximum).

b. FIGO, International Federation of Gynecology and Obstetrics
