# Supplementary figures and images for: Dominant-negative transforming growth factor-β receptor-armoured mesothelin-targeted chimeric antigen receptor T cells slow tumour growth in a mouse model of ovarian cancer
Source: Cancer Immunol Immunother. 2022 Sep 27;72(4):917–28. doi: 10.1007/s00262-022-03290-6 (PMC10025183; doi:10.1007/s00262-022-03290-6)

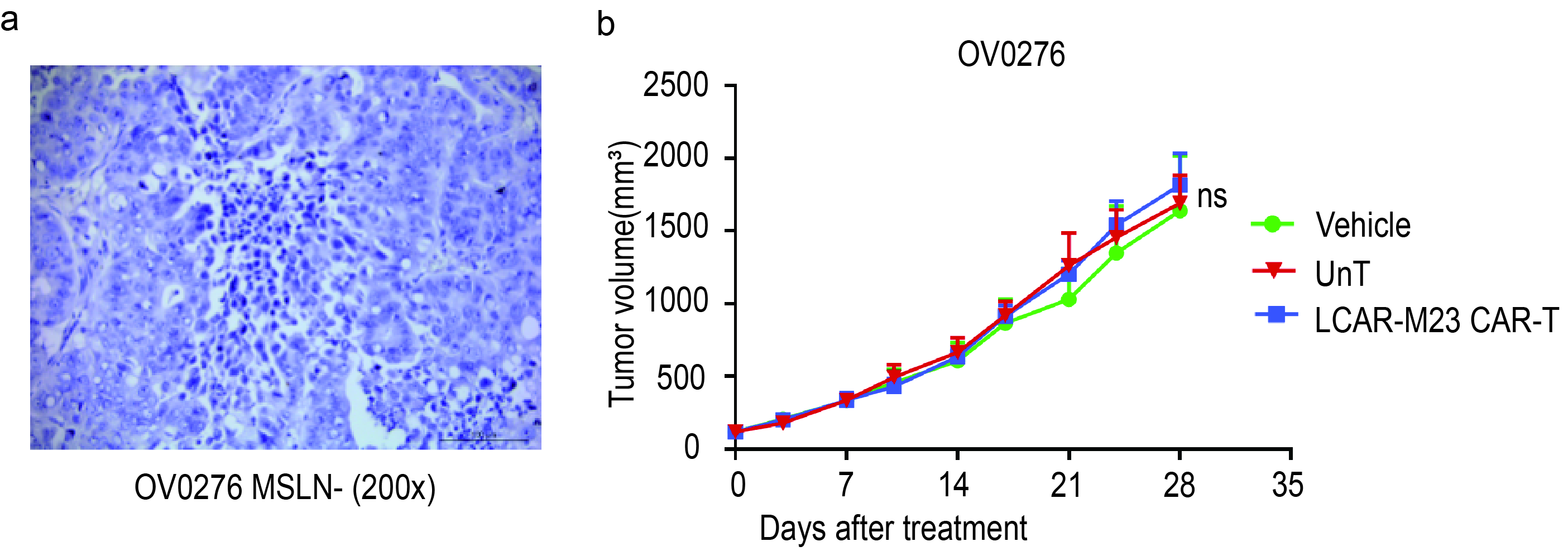

Supplement: Supplementary file 2 — Supplementary file2 (TIF 2494 KB) [file 262_2022_3290_MOESM2_ESM.tif]

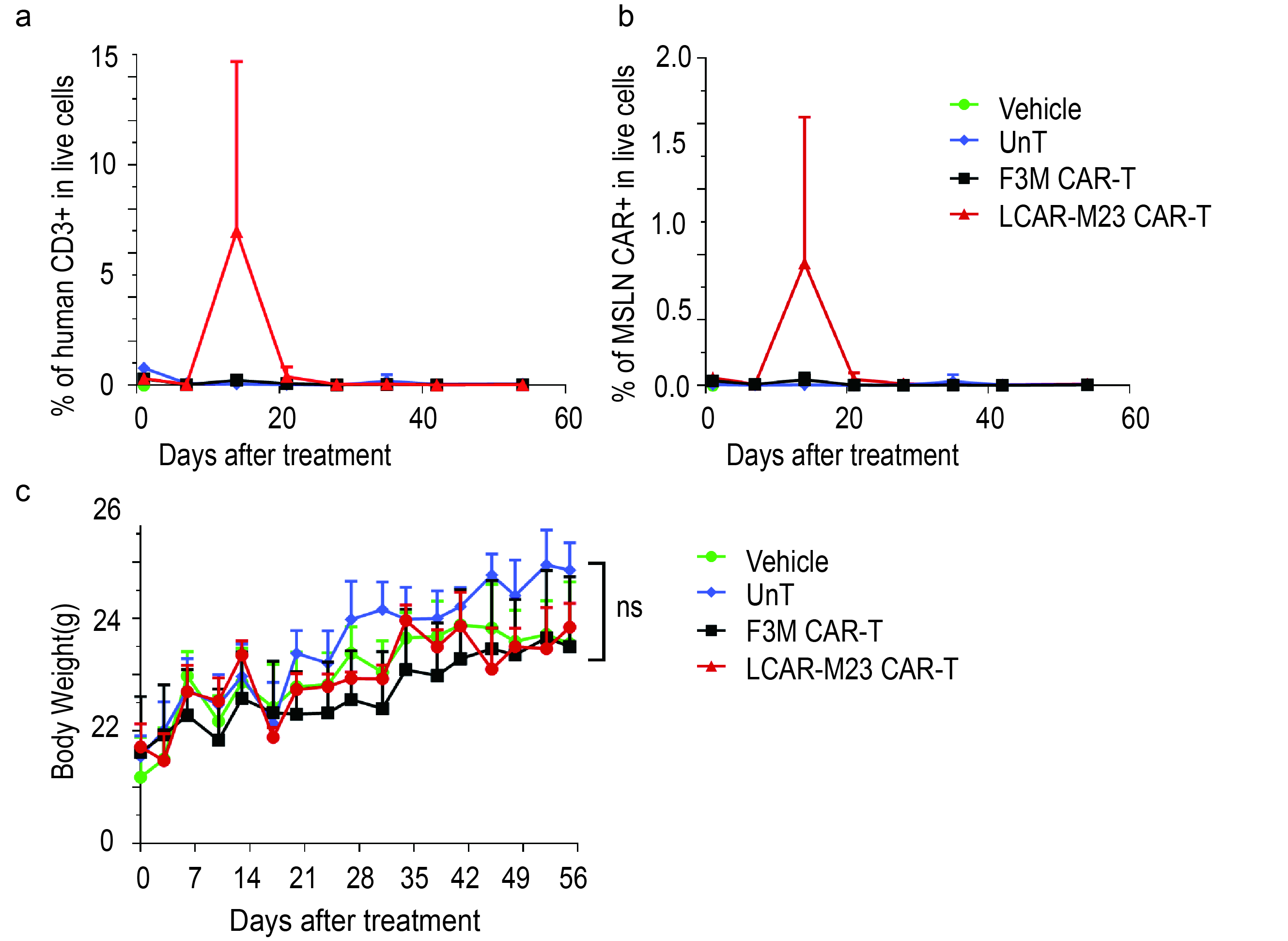

Supplement: Supplementary file 3 — Supplementary file3 (TIF 1244 KB) [file 262_2022_3290_MOESM3_ESM.tif]
